# Location, location, location: Feeding site affects aphid performance by altering access and quality of nutrients

**DOI:** 10.1371/journal.pone.0245380

**Published:** 2021-02-04

**Authors:** Vamsi J. Nalam, Jinlong Han, William Jacob Pitt, Shailesh Raj Acharya, Punya Nachappa

**Affiliations:** 1 Department of Agricultural Biology, Colorado State University, Fort Collins, Colorado, United States of America; 2 Department of Entomology and Plant Pathology, North Carolina State University, Raleigh, North Carolina, United States of America; CSIRO, AUSTRALIA

## Abstract

Aphid feeding behavior and performance on a given host plant are influenced by the plants’ physical and chemical traits, including structural characters such as trichomes and nutritional composition. In this study, we determined the feeding behavior and performance of soybean aphids (*Aphis glycines*) on the stem, the adaxial (upper), and the abaxial (lower) leaf surfaces during early vegetative growth of soybean plants. Using the electrical penetration graph technique, we found that aphids feeding on the stem took the longest time to begin probing. Once aphids began probing, the sieve elements were more conducive to feeding, as evidenced by less salivation on the stem than either leaf surface. In whole-plant assays, stems harbored higher aphid populations, and aphids had shorter development time on stems than the adaxial and the abaxial leaf surfaces. We compared trichome density and length on the stem, the adaxial, and the abaxial leaf surfaces to investigate whether plant trichomes affected aphid feeding and performance. There were higher density and longer trichomes on stems, which likely resulted in aphids taking a longer time to probe. Still a negative impact on aphid population growth was not observed. Analysis of phloem sap composition revealed that vascular sap-enriched exudates from stems had higher sugars and amino acids than exudates from leaves. In artificial diet feeding assays, the population of aphids reared on a diet supplemented with stem exudates was higher than on a diet supplemented with leaf petiole exudates which is in agreement with results of the whole-plant assays. In summary, our findings suggest that the performance of soybean aphids on a specific plant location is primarily driven by accessibility and the quality of phloem composition rather than structural traits.

## Introduction

Aphid behavior and performance on a given host plant is modulated by the plant’s nutrient composition and defensive secondary metabolites [1–7]. It is expected that insects choose plant parts that are most conducive to their reproductive success, and as a consequence, the most suitable parts harbor the highest densities. For phloem-feeding insects such as aphids, the phloem sap’s nutritional composition is the primary driver of fitness [reviewed in [8]]. Several studies have demonstrated that within-plant variation in sugar and amino acid content of phloem sap influences aphid settling and feeding behavior. Populations of the poplar leaf aphid (*Chaitophorous populicola*) appear to move from leaf to leaf on Eastern cottonwood (*Populus deltoides*), tracking the higher amino acid content in younger or “sink” leaves [9, 10]. Similarly, the green peach aphid (*Myzus persicae*) has been found to move from leaf to leaf as their host plant develops, tracking “sink” leaves [11]. The nutritional quality of the phloem sap also affects aphids’ performance and abundance. For example, green peach aphid and potato aphid (*Macrosiphum euphorbiae*) performed better on developmentally young potato (*Solanum tuberosum*) plants with high glutamine levels than on mature plants with low glutamine levels [12].

Plant morphology can also have a strong influence on aphid behavior and performance [4, 6, 13–15]. Soroka and Mackay [15] found that fecundity, survival, and longevity of the pea aphid (*Acyrthosiphon pisum*) is reduced on a leafless cultivar where leaflets are substituted by tendrils when compared to normal leaf cultivars. In contrast, leaf size did not affect pea aphid fecundity, but longevity was significantly influenced by leaf type and stipule size [16]. Plant trichomes can confer physical resistance by impeding insect movement, feeding, and chemical resistance by exuding noxious secondary metabolites [13, 14]. Trichomes can also impact herbivores by positive [17] or negative [18] effects on natural enemy populations. Dai *et al*. [19] found that trichome density positively affected the abundance of some herbivores and predators but did not affect the density of soybean aphids (*Aphis glycines*). These studies highlight the importance of feeding location on aphid fitness.

The soybean aphid, a phloem-feeding insect native to Asia, was first detected in the U.S. in 2000 and is ubiquitous in all soybean (*Glycine max*) growing regions in the U.S. [20, 21]. In addition to direct damage due to feeding, soybean aphids are competent vectors of many economically important plant viruses [21]. Under ideal conditions, soybean aphid populations can double in 1.5 days, and a single plant can harbor thousands of aphids [22]. A suite of Integrated Pest Management (IPM) strategies, including prophylactic neonicotinoid seed treatment, development of economic thresholds and injury levels, and deployment of aphid-resistant cultivars, have been successful in controlling soybean aphids [23]. However, the best option for control remains to scout and apply foliar insecticides when an economic threshold of 250 aphids/plant is reached [24]. Hence, from a practical standpoint, knowledge of the within-plant distribution of aphids is vital for accurate and rapid estimation of population densities. Within the plant, the distribution of soybean aphids varies with time. Early in the season, aphids are found on young leaves, higher in the canopy, and as the season progresses, aphids move lower in response to predation [25, 26]. A previous study reported that soybean aphids are most often found on the undersides of leaves [20]. However, there is no information on factors underlying soybean aphid’s occurrence on specific plant locations.

The current study aims to investigate how plant location affects host plant acceptance and suitability for soybean aphids. To this end, we investigated soybean aphid feeding behavior on the stem, the adaxial (upper), and the abaxial (lower) leaf surfaces of soybean plants using the electrical penetration graph (EPG) technique and determined aphid performance on the different plant locations. Further, we examined plant traits, such as trichome length and density, and phloem sap nutritional composition as possible mechanisms underlying the specific plant location’s suitability. Lastly, we performed feeding assays with aphids reared on an artificial diet supplemented with vascular sap enriched stem and leaf petiole exudates.

## Materials and methods

### Plant and insect maintenance

The soybean variety Asgrow^®^ AG3432 (Bayer Crop Science, Kansas City, MO), devoid of any seed treatment (naked seed), was used for all experiments and aphid colony maintenance. Plants were grown in Mastermix^®^ 830 soilless media (Mastermix, Quakertown, PA) in 15.24 cm diameter x 14.61 cm height plastic pots and watered three times per week. Plants were fertilized using Miracle Gro (Scott’s Company, Marysville, OH) solution once per week. The plants were maintained at 60–70% relative humidity, temperature of 24 ± 1 °C, and a photoperiod of 16:8 (L:D) hours (h) at photosynthetically active radiation (P.A.R.) of 460 μmol/m^2^/sec in an environmental chamber. All experiments were initiated when plants were at the V1 stage. At this stage, plants had a fully developed trifoliate leaf at the node above the unifoliate nodes based on the phenology scale described in Ritchie et al. [27]. The lab colony of soybean aphids was started from a population (~100–200 individuals) of all life stages collected initially from soybean fields at the Pinney Purdue Agricultural Center, Wanatah, Indiana. In the laboratory, the aphid colony was maintained on AG3432 plants at the V2 to V4 stage at a temperature of 24 ± 1°C and a photoperiod of 16:8 (L:D) h in a 30 × 30 × 76 cm insect cage (BioQuip, Rancho Dominguez, CA). The colony was replenished with fresh plants at the V1 to V4 stage as and when needed or approximately every 4–5 days. Adult apterous aphids were used for all assays except for artificial diet feeding assays, where fourth instar nymphs were used.

### Electrical penetration graph (EPG) analysis

Aphid feeding behavior on the abaxial, the adaxial leaf surfaces, and the stems of soybean plants were monitored using the electrical penetration graph (EPG) [28] technique on a GIGA 8 complete system (EPG Systems, Wageningen, Netherlands) [29]. The EPG experiments were performed on plants at the V1 stage, and recordings were performed on aphids confined to specific plant locations on individual whole plants. Soybean leaflets from the first trifoliates were chosen as the sites for the adaxial and the abaxial leaf surfaces. The portion of the stems between the first and second trifoliates was selected as the site for stem assays. Apterous adult soybean aphids were starved for one h before wiring. The wired plant electrodes were then placed into the soil, and insect probes adjusted, allowing for contact between the plant surface and the insect. Individual aphids were allowed to feed for eight hours while feeding behavior was recorded. Plants and aphids were discarded after each eight-hour recording. Each feeding experiment was analyzed to determine the amount of time spent in each of the four main phases: pathway or probing phase (C), non-probing phase (NP), sieve element phase (SEP), and xylem phase (G). Parameters that indicate aphid health [30] including the number of probes, total time spent in probing, time to 1^st^ probe, time to first potential drop (pd), the average and total duration of pds, and the number of pds were determined. Parameters associated with xylem feeding, including the number of aphids showing G, time to 1^st^ G, the mean and total duration of time spent in G were measured. Additional parameters associated with the SEP, including time in SEP, the number of phloem salivation (E1) waveforms, time to 1^st^ E1, the total duration of E1, the number of single E1, the total duration of single E1, the number of probes after 1^st^ E1, the total duration of E1 followed by phloem ingestion (E2), the number of E2, the total duration of E2 and time to 1^st^ E2 were also determined. EPG results were analyzed using Stylet+ software (EPG Systems, Wageningen, Netherlands). There were 30 replicates for the adaxial, 33 replicates for the abaxial, and 25 replicates for the stem surface. Data for all three treatments were collected simultaneously over one month. Recordings of aphid feeding that did not show any feeding events and recordings that contained greater than 70% of the recording time in NP + derailed stylet phase (F) + G were not included in our analysis. A behavioral kinetogram was constructed based on the number of transitional events for each waveform [31]. The circles’ area represents the aphid’s percent duration in each waveform (see Results). The arrows indicating transitions between the different waveforms were generated by dividing each waveform’s transitional events by the total number of transitional events.

### Performance assay

Aphid population growth on the adaxial, the abaxial, and the stem surfaces was evaluated by confining ten adult apterous aphids at the specific plant location using foam clip cages (36.5 x 25.4 x 9.5 mm) with a no-thrips screen (BioQuip, Rancho Dominguez, CA). The day aphids were placed on the plant surface was considered day 0, and the total number of nymphs and adult aphids recorded daily for four days. A previous study by McCornack *et al* [22] reported that it takes approximately 2 days for soybean aphid populations to double in size; hence, we allowed 4 days to ensure sufficient population growth. Soybean leaflets from the first trifoliate were chosen as the sites for the abaxial and the adaxial leaf surfaces. The portion between the first and second trifoliates was selected as the site for the aphid performance assay on stems. The aphid population growth on the three different plant locations was measured on separate whole plants. The experiment was repeated on three independent occasions over three months, with five replicates or plants per treatment per experiment, for a total of 15 replicate plants per treatment.

To determine whether an increase in population growth on the stem was due to shortened development time or increased adult longevity, separate experiments were conducted on whole plants. A single apterous adult soybean aphid was confined to the adaxial, the abaxial and the stem surfaces using clip cages on whole plants. We used separate plants for each plant location. Adults were allowed to reproduce for 48 h and then removed. The offspring were confined in the clip cage in the same location until the end of experiment (no live aphids). The cohort of offspring produced was monitored daily, and development time or time to reach adulthood and adult longevity were recorded. For the entire duration of the experiment, offspring were confined to the same location using clip cages. The area available for aphids on the adaxial and the abaxial surfaces was equal, but the experimental setup’s limitations created different surface areas for aphids confined to the stems. However, there was ample room and resources for aphids in all setups to reproduce, feed, grow, and survive, so we did not account for surface area in the analyses. We performed three independent experiments over three months, with five replicates or plants per treatment per experiment, for a total of 15 replicate plants per treatment.

### Trichome measurements

The number and length of trichomes on fully-expanded first and second trifoliate leaves and stems were determined using a digital camera connected to a Leica Zoom 2000 dissection microscope. Trichome lengths were measured using Image J (https://imagej.nih.gov/ij/). Trichome numbers between veins (interveinal) were counted on area/leaf section of 1 cm^2^ on the adaxial and the abaxial surfaces on six randomly chosen areas on the first and second trifoliate leaves. For the stem, trichome numbers were calculated in an area of 3.5 mm^2^ from four randomly chosen areas on the stem between the first and second trifoliate leaves. All measurements were performed on independent plants. Trichome lengths on the adaxial and the abaxial leaf surfaces and stems were measured on 20 randomly chosen trichomes per location per plant. Experiments were conducted with six replicates per treatment per experiment for 18 replicate plants per treatment. To count the trichome number along the veins, we followed the same procedure described above. The length of trichomes along the veins on both leaves was measured from 25 randomly chosen trichomes per plant with four replicates, for a total of 12 replicate plants per treatment. All the experiments mentioned were performed on three independent occasions over three months.

### Collection of leaf petiole and stem exudates

In separate experiments, plants (variety AG3432) were grown in pots with a 30.5 cm diameter (Myers Industries Marysville, OH, U.S.A.) until the V1 growth stage. Plants were grown in the same conditions and fertilized, as mentioned previously. Vascular sap-enriched stem and leaf petiole exudates were collected as per Nachappa *et al*. [32]. To prevent bacterial contamination, trifoliates were cut at the base of the petioles or stems cut two cm above the soil surface, were immersed in 50% ethanol, and then immediately moved to 0.05% bleach solution for no more than 2–3 seconds to achieve surface sterilization of the cut surfaces. The cut trifoliates and stems were then transferred to 1 mM EDTA solution (pH 8.0) until sampling was completed. Before placing the cut trifoliates and stems in 1 mM EDTA for overnight exudation, an additional one cm section above the previously cut surface for both the petioles and stems was cut. Three petioles and/or stems with the trifoliates still attached were weighed (to obtain the fresh weight of the sample) before being placed into the single well of a 6-well plate containing 6 mL of 1 mM EDTA. After completing the transfer of all samples, the entire setup (plant samples in 6-well plate) was placed in a terrarium with a clear lid lined with moistened paper towels for 24 h. At the end of the exudation period of 24 h, vascular sap-enriched stem and petiole exudates from three wells were then pooled per sample resulting in nine stems/petioles per pooled sample. Samples were then filtered through 0.2 μm pore size filters and lyophilized. After lyophilization, samples were eluted in 750 μL of 1mM EDTA. In total, leaf petiole and stem exudates were collected from 54 independent plants. Exudates from nine plants (stems or leaves) were pooled to form one composite sample; hence a total of six composite/pooled samples were submitted for gas chromatography-mass spectrometry analysis (GC-MS) analysis and used in the artificial feeding assays.

### Artificial feeding assay

An artificial diet developed for soybean aphids [33] was supplemented with the lyophilized and reconstituted stem or leaf petiole exudates and used in artificial feeding assays. An artificial feeding chamber consisted of 55 mm Petri dishes (V.W.R., Radnor, PA) with parafilm (Bemis, Neenah, WI) sachets containing 750 μL of diet. The diets were supplemented with 50 μL of leaf petiole exudates or 50 μL of stem exudates. Ten fourth instar aphid nymphs were placed in each feeding chamber and allowed to mature and reproduce. The total number of nymphs and adults were counted every day for four days. The assay was conducted under laboratory conditions at ambient temperatures of 24 ± 1°C and 16:8 (L:D) h. The experiment was conducted on three independent occasions over three months—the first two experiments had five replicates or artificial feeding chambers per treatment. The third experiment had only four replicates per treatment.

### Analysis of leaf petiole and stem exudates

Sucrose and amino acid content of the leaf petiole and stem exudates was quantified by GC-MS The samples arranged in a randomized order were extracted and injected to GC-MS along with three quality checks that were generated by combining a small aliquot of each sample. For sucrose quantification, samples were first diluted by mixing 50 μL of the sample with 450 μL of water. Then 50 μL of the diluted sample was mixed with 200 μL of 50% methanol in water containing 25 μg/mL of internal standard (D-Sucrose-13C12, 98%, Cambridge Isotope Laboratories, Inc.). After samples were well mixed, 30 μL were aliquoted and dried under nitrogen. For amino acids quantification, samples were first diluted by mixing 80 μL of the sample with 80 μL of 100% methanol. Then 3.2 μL of internal standard mix containing 200 μg/mL of L-^13^C_4_-asparagine (99%, Cambridge Isotope Laboratories, MA), ^13^C_6_,^15^N_2_-L-lysine (99 atom % ^15^N, 99 atom % ^13^C, 95% (CP), Sigma-Aldrich), threonine-^13^C_4_,^15^N (98 atom % ^13^C, 98 atom % ^15^N, Sigma-Aldrich), leucine-d_10_ (98 atom % D, Sigma-Aldrich), tryptophan-d3 (Santa Cruz Biotechnology) was added to each sample. After thorough mixing, 100 μL were aliquoted, dried under nitrogen, and then stored until analysis. The analysis of the dried samples was carried out at -80°C as per Walsh *et al*. [34]. Briefly, the dried samples were resuspended in 50 μL of pyridine containing 25 mg/mL of methoxyamine hydrochloride and incubated at 60°C for 45 min. The samples were then vigorously vortexed for 30 s, sonicated for 10 min, and incubated for an additional 45 min at 60°C. Next, 50 μL of N-methyl- N-trimethylsilyltrifluoroacetamide with 1% trimethylchlorosilane (MSTFA + 1% TMCS, Thermo Scientific) were added, and samples were vigorously vortexed for 30 s, incubated at 60 °C for 30 min. Metabolites were detected using a Trace 1310 GC coupled to a Thermo ISQ mass spectrometer. Samples (1 μL) were injected at a 10:1 split ratio to a 30 m TG-5MS column (Thermo Scientific, 0.25 mm i.d., 0.25 μm film thickness) with a 1.2 mL/min helium gas flowrate. GC inlet was held at 285°C. The oven program started at 200°C for 30 s, followed by a ramp of 15°C/min to 330°C, and a 4 min hold. Masses between 50–650 m/z were scanned at five scans/sec under electron impact ionization. The transfer line and ion source were held at 300 and 260°C, respectively. Pooled QC samples were injected after every six actual samples.

### Statistical analysis

All data were tested to determine if they conform to the assumptions of analysis of variance (ANOVA) using the Anderson-Darling statistic in Minitab 19. The aphid feeding behavior data from the EPG experiments were rank transformed, following which a one-way ANOVA was used to determine the differences between groups. Tukey’s post-hoc test was used to determine differences between means at a *P-*value of ≤ 0.05. The aphid counts in the population growth assays conformed to assumptions of ANOVA, and no transformations were needed. A mixed-model ANOVA was used to assess the effects of feeding location on the total number of nymphs on day 4. We also included a random block effect to account for the variation in experimental repetition (random block of time). The development time and adult longevity data did not conform to ANOVA assumptions after transformations; hence a non-parametric Kruskal-Wallis test was performed. The trichome measurements were rank transformed, after which a mixed-model one-way ANOVA with fixed effects (treatments) and random effects (experiment) was performed. The artificial feeding assay data were rank transformed and analyzed using a mixed-model one-way ANOVA with fixed effects (treatments) and random effects (experiment). A two-sample t-test was performed to compare the mean quantities of sucrose and total free amino acids from the stem and leaf petiole exudates. All statistical analyses were performed using Minitab Version 19 (Minitab, Stat College, PA).

## Results

### Aphid feeding behaviors

We performed an electrical penetration graph (EPG) analysis of aphid feeding behaviors on the three host locations.

#### Probing behavior

The time from the start of the EPG recording to the first probe varied significantly among aphids feeding on the three host locations (Table 1). Aphids feeding on the stem took the longest to begin probing, whereas aphids feeding on the abaxial leaf surface took the shortest time to the first probe (Table 1). Aphids feeding on the stem also showed a significantly lower total number of probes and short probes (i.e., probes lasting less than 3 min) (Table 1) as compared to aphids feeding on either leaf surface. Although the number of probes by aphids feeding on all three host locations decreased during EPG recordings, aphids on the adaxial leaf surface displayed higher probing during the fourth and fifth hour of EPG recording as compared to aphids feeding on the abaxial or the stem (S1 Fig, *P* < 0.001).

**Table 1:** Non-phloem feeding behaviors of aphids feeding on the adaxial, the abaxial and the stem surfaces of soybean plants.

Parameters	Adaxial (Upper) ^a^	Abaxial (Lower) ^a^	Stem ^a^	*P-value* ^b^	*F-value*^c^
	N = 25	N = 30	N = 33		
***Probing Behavior***					
Time spent in non-probing (min)	137.1 ± 22.6 b	199.3 ± 27.7 ab	288.2 ± 34.1 a	**0.003**	F_2,86_ = 6.33
Time to 1^st^ Probe (min)	7.5 ± 2.3 b	3.7 ± 1.7 b	8.7 ± 2.5 a	**0.005**	F_2,86_ = 5.35
Number of probes	29.1 ± 2.9 a	23.4 ± 2.6 a	12.0 ± 2.3 b	**<0.0001**	F_2,86_ = 14.63
Number of short probes (C<3 min)	18.9 ± 2.2 a	15.7 ± 1.9 a	8.2 ± 1.8 ab	**<0.0001**	F_2,86_ = 12.47
Total probing time (min)	342.8 ± 22.6 a	280.6 ± 27.7 ab	191.4 ± 34.2 b	**0.003**	F_2,86_ = 6.33
Total duration of C (min)	210.3 ± 17.7 a	128.8 ± 15.5 b	75.0 ± 13.7 c	**<0.0001**	F_2,86_ = 16.35
Number of potential drops (P.D.)	136.0 ± 12.3 a	80.5 ± 10.6 b	35.7 ± 7.3 c	**<0.0001**	F_2,86_ = 21.43
Average duration of PD (min)	0.14 ± 0.01	0.13 ± 0.01	0.25 ± 0.11	0.873	F_2,86_ = 0.873
Sum duration of PD (min)	17.6 ± 10.4 a	10.9 ± 2.1 b	17.7 ± 1.7 a	**<0.0001**	F_2,86_ = 11.94
***Xylem Feeding***					
Aphids with xylem phase %	90 (27/30)	75.8 (25/33)	48 (12/25)		
Number of G	11.4 ± 1.5	9.4 ± 1.3	7.3 ± 1.2	0.502	F_2,62_ = 1.32
Time to 1^st^ G (min)	66.6 ± 20.8	62.7 ± 15.8	49.2 ± 8.2	0.715	F_2,62_ = 0.34
Mean duration of G (min)	11.4 ± 3.2	25.9 ± 9.5	39.1 ± 9.1	0.78	F_2,62_ = 0.25
Time spent in G (min)	75.5 ± 15.7	104.8 ± 15.3	82.5 ± 14.5	0.21	F_2,62_ = 0.59

One-way ANOVA was used to analyze the effects of treatments on each parameter.

^a^ Data presented are the means ± standard error of mean for aphids that displayed the behaviors.

^b^ P-values that are significant are highlighted in bold.

^c^ The degrees of freedom for the xylem feeding phase is 62 since not all aphids displayed xylem phase.

#### Potential drops

Within the pathway phase (also referred to as the probing phase, C), potential drops (pd) are found, which represent intracellular punctures made by stylet tips [35]. These penetrations play an essential role in host selection since they allow the aphid to sample cell contents. The total number of pd was lowest on stems, followed by the abaxial and the adaxial leaf surfaces (Table 1). The total time spent by the aphids in pd was shortest on the abaxial leaf surface. However, the time spent by the aphids in sampling cell contents, i.e., the average time spent in pd on the three host locations, was not significantly different (Table 1).

#### Xylem phase (G)

**Fig 1:**
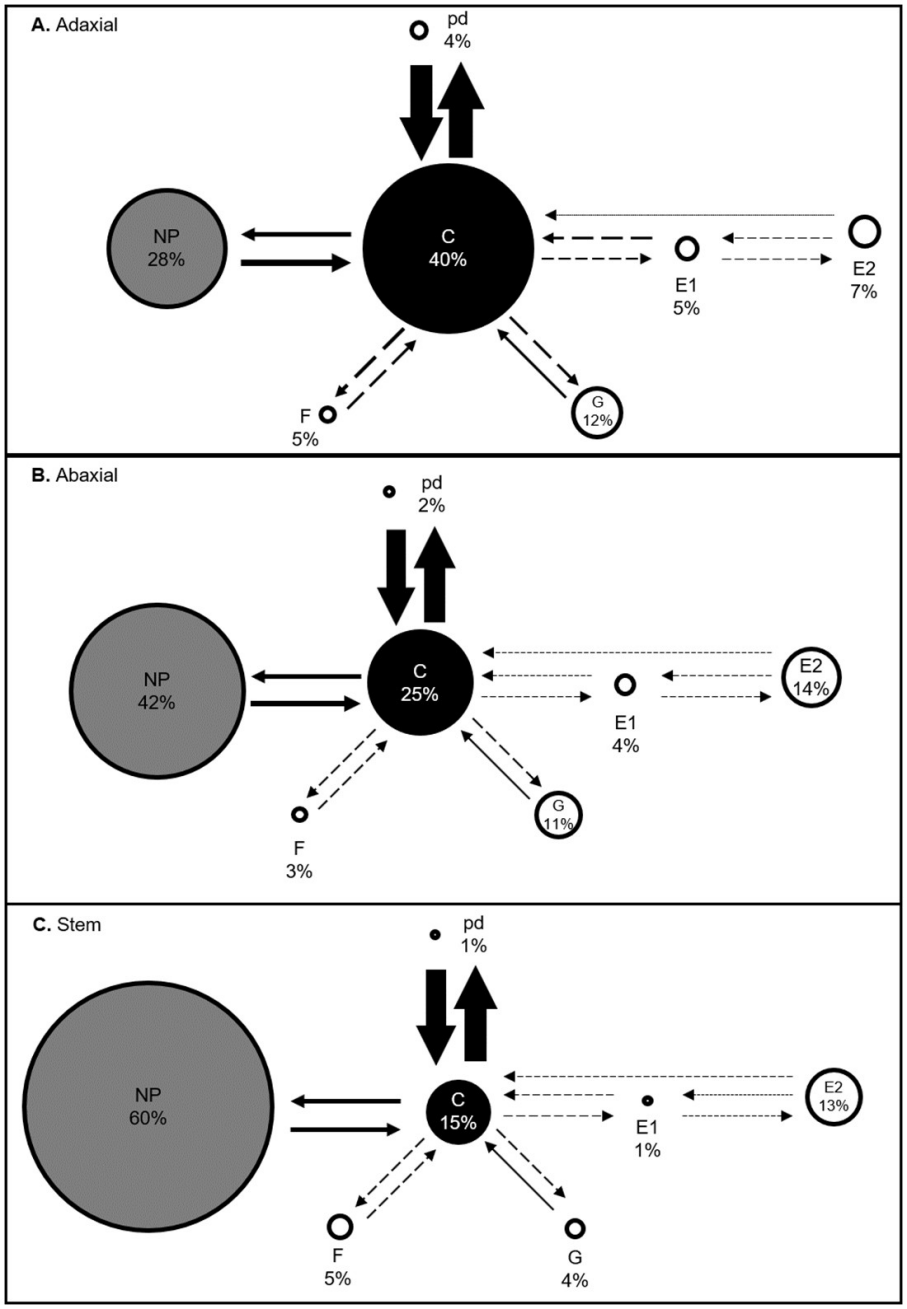
Behavioral kinetogram of aphid feeding behaviors. Circle areas represent the proportion of time spent by soybean aphids in different behaviors on the three different plant locations: (A) the adaxial, (B) the abaxial, and (C) the stem. Arrows represent transitions with arrow thickness proportional to frequency. Values are averages of 30, 33, and 25 samples for the adaxial, the abaxial, and the stem surfaces, respectively. The time spent by the aphids in various activities (NP, non-probing phase; C, pathway phase; pd, potential drops; F, derailed stylets; E1, phloem salivation; E2, phloem ingestion; G, xylem phase) was analyzed by using rank transformation followed by one-way ANOVA. The circles’ different colors indicate values that are significantly different (*P* < 0.05) from each other. Solid arrows represent values that are significantly different (*P* < 0.05), and dashed arrows represent values that are not significantly different from each other.

The active ingestion of xylem sap is an essential mechanism for the rehydration of aphids [36, 37]. There were no significant differences in time spent in G by aphids feeding on the three locations (Fig 1A–1C). However, a larger percentage of aphids feeding on the adaxial leaf surface entered the xylem phase compared to the abaxial leaf surface and stem (Table 1). Additional parameters related to the xylem phase of aphid feeding were evaluated, including the number of G, the time to first G, and the mean duration of G, and no significant differences were observed for any of these parameters (Table 1).

#### Sieve element phase (SEP)

The SEP occurs when the stylet is located in the sieve element [38]. The SEP consists of the E1 phase during which salivation into the sieve element occurs and the E2 phase, correlated with passive ingestion of phloem sap and concurrent salivation. Aphids can also display a single E1 phase that is not followed by E2 (phloem ingestion), indicating the aphid’s inability to continuously feed from the sieve element and enter into E2. Among aphids feeding on the stem, a smaller number displayed SEP than aphids feeding on the adaxial and the abaxial leaf surfaces, respectively (Table 2). Considering only the aphids that fed from the sieve element on the three host locations, a significant difference in total time spent in SEP was not observed (Fig 1A–1C, Table 2). A closer examination of aphid behavior in SEP shows significant differences in several parameters related to the E1 phase. The number of E1 and single E1 (i.e., E1 waveforms without a subsequent E2 phase) was significantly lower in the stem and abaxial leaf surface than the adaxial surface (Table 2). Aphids on the stem also spent a significantly lower amount of time in the E1 phase (Table 2). The total duration of time spent in single E1 was the highest in the aphids feeding on the adaxial leaf surface and was significantly lower on the abaxial surface and stem (Table 2). Additionally, aphids feeding on the stem exhibited a reduced number of probes once they had successfully located and began feeding from the sieve element (Table 2). Concerning the E2 phase (phloem ingestion), no significant differences were observed for any of the parameters measured. Although not statistically significant, aphids did seem to spend a longer time in the E2 phase on stems compared to the adaxial and the abaxial leaf surfaces (Table 2).

**Table 2:** Phloem feeding behaviors of aphids feeding on the adaxial, the abaxial, and the stem surfaces of soybean plants.

*Sieve Element Phase*	Adaxial (Upper)^a^	Abaxial (Lower) ^a^	Stem ^a^	*P-value*^b^	*F-value*_(2,63)_^c^
	N = 25	N = 30	N = 33		
Aphids with SEP (%)	80 (24/30)	81.8 (27/33)	60 (15/25)		
Time in SEP (min)	73.0 ± 14.8	105.6 ± 25.4	112.8 ± 36.5	0.98	0.02
Number of E1 waveforms	9.0 ± 2.8 a	3.9 ± 0.5 ab	3.5 ± 1.4 b	**0.012**	4.76
Time to 1^st^ E1 (min)	144.8 ± 18.8	125.4 ± 18.1	167.9 ± 32.8	0.206	0.61
Total duration of E1 (min)	30.3 ± 5.6 a	24.6 ± 8.7 ab	8.3 ± 2.5 b	**0.014**	4.54
Number of single E1	5.6 ± 2.5 a	1.5 ± 0.4 b	1.6 ± 1.2 b	**0.002**	6.74
Total duration of single E1 (min)	12.6 ± 3.1 a	6.5 ± 2.3 ab	3.6 ± 2.4 b	**<0.0001**	10.06
Number of probes after 1st E	13.5 ± 1.9 a	11.3 ± 1.9 a	2.2 ± 0.4 b	**<0.0001**	8.61
Total duration of E1 followed by E2 (min)	68.8 ± 14.6	97.3 ± 26.1	136.5 ± 38.5	0.99	0.20
Number of E2 waveforms	3.4 ± 0.5	2.3 ± 0.4	2.3 ± 0.3	0.42	0.43
Total duration of E2 (min)	51.2 ± 13.7	80.9 ± 22.5	130.5 ± 38.3	0.092	0.41
Time to 1^st^ E2 (min)	199.4 ± 17.2	134.1 ± 17.3	198.8 ± 41.3	0.06	2.95

One-way ANOVA was used to analyze the effects of treatments on each parameter.

^a^ Data presented are the means ± standard error of mean for aphids that displayed the behaviors.

^b^ P-values that are significant are highlighted in bold.

^c^ The degrees of freedom for the sieve element phase is 63 (n = 66) since not all aphids displayed sieve element phase.

A behavioral kinetogram was constructed based on the transitions to and from each waveform and representing the duration of time spent in each waveform (Fig 1, S1 Table).

### Population growth of aphids

**Fig 2:**
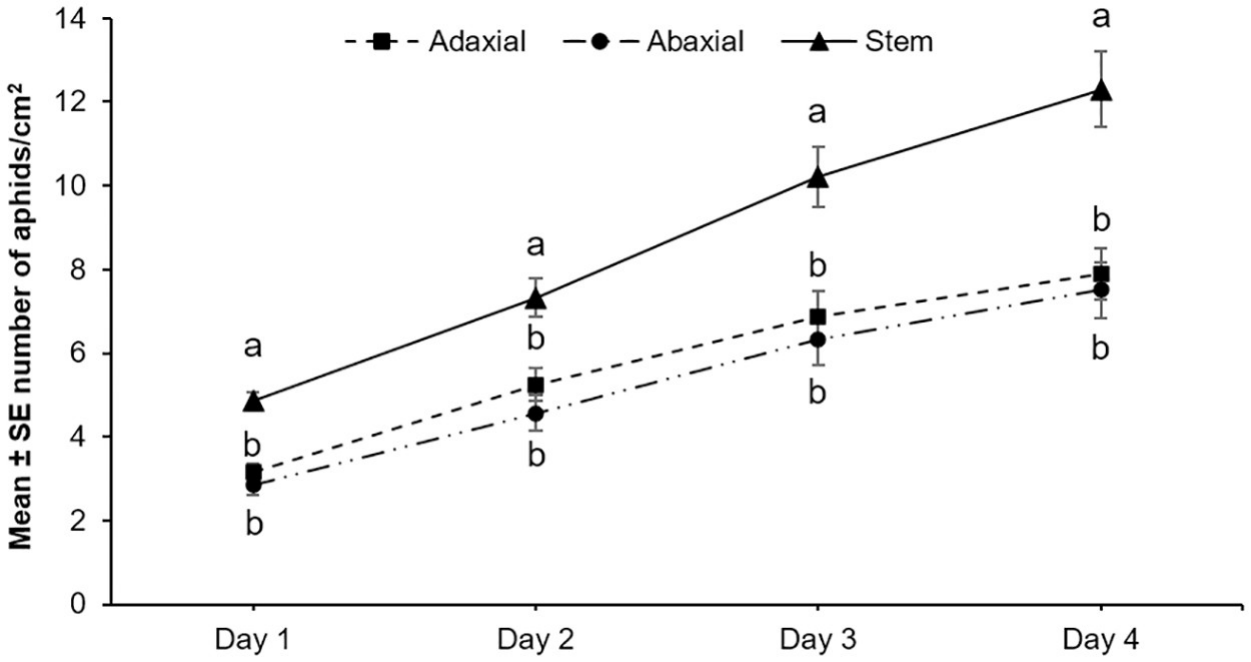
Aphid performance is enhanced on stems. Population growth of aphids on the adaxial, the abaxial, and the stem surfaces of soybean plants. The numbers of adults and nymphs were recorded daily for four days. Five replications were performed for each plant location, and the experiment was repeated three times. Different letters above the bars indicate values that are significantly different from each other (*P* < 0.05).

To determine aphids’ performance on different plant parts, the population of aphids, that is, the total number of nymphs and adult aphids were measured daily for four days. The aphid population significantly differed between plant locations on day 4 (F_2,40_ = 25.09, *P*<0.001). Soybean stems supported a significantly greater population of aphids than either leaf surface over the experiment (Fig 2). There was no effect of block or experimental repetition on aphid populations (*P* = 0.171). The development time or time to reach adulthood was significantly shorter on stems than on either the adaxial or the abaxial leaf surfaces (Table 3). Feeding location, on the other hand, did not influence adult longevity (Table 3).

**Table 3:** Development time and longevity of soybean aphids reared on different plant locations.

	Adaxial	Abaxial	Stem	*P*-value^a^	H-value^b^
Time to adulthood (days)	8.1 ± 0.3 a	8.4 ± 0.3 a	6.9 ± 0.3 b	**0.001**	12.18
Longevity (days)	11.2 ± 0.8	12.0 ± 0.7	13.1 ± 0.4	0.208	10.14

Kruskal-Wallis non-parametric test was used to analyze the effects of treatments on each parameter.

^a^*P*-values that are significant are highlighted in bold.

^b^H-value is the Kruskal-Wallis test statistic.

### Trichome density

There were significant differences between the three plant locations for trichome density and length (Table 4). The density of trichomes on the stem was significantly higher than on both leaf surfaces, with the stems having 8.4 times and 6.5 times the densities on the adaxial and the abaxial surfaces, respectively (F_2,45_ = 62.4, *P <* 0.001). The abaxial leaf surface had a significantly higher density of interveinal trichomes than the adaxial leaf surface. A significant difference was observed with trichome lengths, with trichomes on the stem measuring twice as long as trichomes on either leaf surface (F_2,177_ = 49.43, *P* < 0.001). There was no effect of block or experimental repetition on trichome density (*P* = 0.809) and length (*P* = 0.361). The density (F_1,34_ = 102.32, *P* < 0.001) and length (F_1,298_ = 5.48, *P* < 0.020) of trichomes along the veins were significantly higher on the abaxial leaf surface as compared to the adaxial leaf surface. Block effects on trichome density and length along the vein were not significant (*P* = 0.329 and *P* = 0.109, respectively).

**Table 4:** Mean ± S.E. trichome densities on the adaxial, the abaxial and the stem surfaces of soybean plants.

Location of trichomes	Adaxial ^a^	Abaxial ^a^	Stem^a^
Number of trichomes (per mm^2^)	3.29 ± 0.33 c	4.24 ± 0.34 b	27.60 ± 1.75 a
Length of trichome* (mm)	7.03 ± 0.37 b	6.64 ± 0.42 b	12.68 ± 0.62 a
***Veins***			
Number of trichomes (per mm)	0.16 ± 0.01 b	0.39 ± 0.01 a	n.a.
Length of trichome* (mm)	0.69 ± 0.03 b	0.76 ± 0.02 a	n.a.

A mixed-model ANOVA was used to analyze the effects of treatments on each parameter.

^a^ Within a row, values followed by the same letter are not different (*P* <0.05).

n.a.–not applicable; no veins on stem.

### Sugars and amino acids

**Fig 3:**
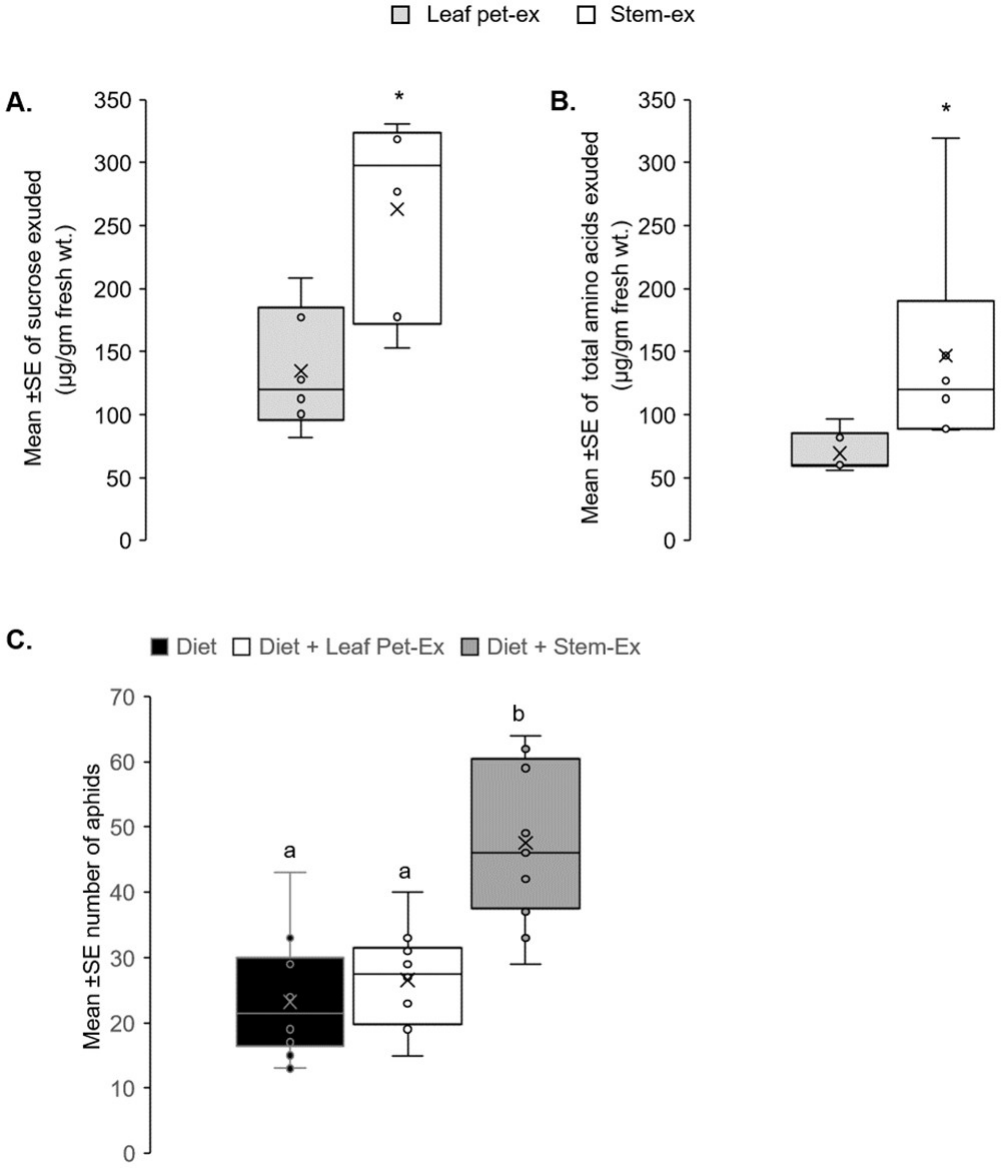
Phloem sap quality of stem supports higher aphid populations. GC-MS was used to determine the amounts of sucrose (A) and amino acids (B) in exudates collected from the stem and leaf petiole exudates. Artificial diet assay with stem and petiole exudates (C). Comparison of aphid numbers on an artificial diet supplemented with appropriate amounts of stem and leaf petiole exudates. Control was an artificial diet alone (diet). Aphid numbers were monitored for four days, and data for day four is presented. In (A) and (B), asterisks indicate significant differences (*P* < 0.05) (*). For (C), different letters above the bars indicate values that are significantly different (*P* < 0.05) from each other.

Given that aphids had higher population growth and shorter development time on stems, we sought to determine if differences in phloem sap composition between the stems and leaves contributed to the observations. Vascular sap-enriched stem and leaf petiole exudates analyzed using GC-MS showed that the quantity of sucrose and total free amino acids is significantly higher in the stem-exudates than leaf petiole exudates (t = -3.42; *P* = 0.009 and t = -2.15, *P* = 0.008, respectively; Fig 3A and 3B). Of the 18 amino acids measured, the concentration of 12 amino acids was significantly different (Table 5). Eight of the 12 amino acids- aspartic acid, tyrosine, asparagine, threonine, leucine, alanine, phenylalanine, and proline—were significantly higher in the stem. On the other hand, the concentrations of lysine, tryptophan, serine, and glutamine were significantly lower in the stems than the leaf petiole exudates (Table 5).

**Table 5:** Concentrations of the individual amino acid (ng/gm fresh weight) in stem- and leaf petiole-exudates of soybean plants.

Amino Acids	Leaf petiole^a^	Stem^a^	*P*-value^b^
Alanine	0.12 ± 0.01	0.48 ± 0.1	**0.004**
Arginine	11.28 ± 1.94	20.49 ± 4.76	0.109
Asparagine	2.27 ± 0.31	8.08 ± 0.83	**0.004**
Aspartic Acid	38.08 ± 5.41	93.96 ± 28.84	**0.016**
Glutamic Acid	0.67 ± 0.04	0.61 ± 0.09	0.423
Glutamine	0.21 ± 0.01	0.04 ± 0.01	**0.004**
Glycine	0.3 ± 0.04	0.42 ± 0.09	0.262
Isoleucine	0.92 ± 0.02	0.83 ± 0.11	0.055
Leucine	0.7 ± 0.02	1.47 ± 0.18	**0.004**
Lysine	0.8 ± 0.08	0.43 ± 0.05	**0.004**
Methionine	2.12 ± 0.45	1.14 ± 0.27	0.201
Phenylalanine	0.12 ± 0.03	0.47 ± 0.16	**0.006**
Proline	0.03 ± 0.01	0.35 ± 0.08	**0.004**
Serine	0.22 ± 0.02	0.16 ± 0.02	**0.037**
Threonine	2.26 ± 0.02	3.54 ± 0.36	**0.004**
Tryptophan	3.87 ± 1.05	0.34 ± 0.08	**0.004**
Tyrosine	1.06 ± 0.29	8.33 ± 1.25	**0.004**
Valine	4.69 ± 0.15	5.85 ± 0.52	0.055

A two-sample t-test was performed to compare sucrose and amino acid content between stem and leaf petiole exudates.

^a^ Values represent mean ± standard error of n = 6 replicates.

^b^ *P*-values that are significant are highlighted in bold.

Artificial feeding assays were conducted using diet enriched with either stem exudates, leaf petiole exudates, or diet alone to determine the impact of phloem composition on aphid population growth. The population of aphids reared on stem exudate supplemented artificial diets was two times greater than the artificial diet alone and 1.7 times greater than diet supplemented with leaf petiole exudates (F_2,39_ = 25.24, *P*<0.001; Fig 3C) with no significant effect of the experiment (*P*-value = 0.47). Taken together, these data suggest that stems represent a higher quality food source for aphids.

## Discussion

The feeding location of aphid populations on a given host plant is modulated by nutritional and secondary metabolite composition in the phloem sap, affecting their performance and abundance [1–7]. Moreover, structural traits such as trichomes, surface waxes, and plant cell wall composition can influence aphid feeding preference on particular plant locations [13, 14, 39]. Here, we show that plant location (stem versus the adaxial and the abaxial leaf surfaces) strongly influences aphid feeding behaviors. In aphids, stylet penetration, specifically phloem sieve element penetration, is essential for host plant acceptance and rejection [41]. The first few stylet penetrations are usually confined to the epidermis and are typically brief (<3 min). These short probes are essential indicators for gustatory cues as aphids ingest small amounts of plant cell contents to determine suitability. We found that soybean aphids show significantly fewer short probes when feeding on stems than the adaxial and the abaxial leaf surfaces. Plant trichomes can act as morphological barriers interrupting and slowing down feeding by herbivores [13, 14, 40]. We found that non-glandular trichomes were denser and longer on stems than either leaf surface, which may have resulted in the reduced number of probes and longer time spent in non-probing observed on the stem. More extended non-probing periods have also been reported for bean aphids (*A*. *fabae*) and bird cherry-oat aphids (*Rhopalosiphum padi*) feeding on stems [41]. However, in the current study, trichomes likely did not negatively impact aphid populations, with stems supporting the highest populations. Moreover, development time was significantly shorter, and the adult aphids lived longer on stems (*albeit* not statistically significant). Similar to our observations, other reports indicate that trichomes did not affect the density of soybean aphids [20, 42]. In contrast to our findings, Tilmon *et al*. [20] reported that soybean aphids are commonly found on the underside (abaxial) of the leaves. Aphid preference for particular plant parts may vary over time throughout the plant’s phenology [43]. Aphid preference for stems has been observed in other aphid-host systems. Prado and Tjallingii [41] report that the stem is the preferred feeding site for bird-cherry oat aphids (*R*. *padi*) on wheat. Berberet *et al*. [44] report similar behaviors for cowpea aphids (*A*. *craccivora*), which exhibited a strong preference for stems as opposed to petioles and leaf blades.

The final behaviors observed during host plant and feeding site selection are phloem acceptance (salivation or first phloem phase, E1) and sustained phloem ingestion (E2 and sE2) [45, 46]. After penetrating sieve elements, aphids secrete watery saliva into the phloem (E1 phase) to suppress phloem-based defenses and allow for prolonged phloem sap ingestion (E2 phase). In aphids feeding on stems, we observed that the total number of E1 waveforms and the time spent in E1 was significantly lower on stems. Watery saliva contains several proteins, some of which have a well-known biochemical activity that could either act as elicitors or suppressors of plant defense [47, 48]. The shorter salivation periods observed in aphids on stems suggest that plant defense responses and phloem-based defenses in stems are reduced compared to leaf tissues. A recent study showed that concentrations of specialized metabolites were high in exudates of stems. Yet, stems supported higher aphid populations, suggesting that aphids can adapt to these compounds [49].

A limitation of our experimental design is that experiments were initiated when plants were at the V1 stage. It is possible that aphid preference for the stem occurs at the early vegetative stage of plant growth and changes in later vegetative and reproductive stages. Indeed, the within-plant distribution of aphids is dynamic over the season [24]. Soybean aphids have greater reproduction rates during vegetative stages than late reproductive stages, presumably because of decreased amino acid content when plants cease growing and their leaves are senescing [43]. In contrast, more recent studies have shown that the effect of plant growth stage on aphid life-history traits is not as pronounced [50–52]. For example, Rutledge and O’Neil [50] found that soybean aphids’ life history traits showed no difference on different growth stages of soybean plants. It is also important to note that predators [53], intra- and inter-specific competition [43] and abiotic factors such as high temperature and rainfall may influence population growth and within-plant distribution of aphids. Nevertheless, mechanisms driving the within-plant distribution of aphids need further investigation. Future research should focus on soybean aphid preference and performance in both laboratory and field conditions and over longer temporal scales to elucidate any potential density-dependent changes in preference and performance, in addition to identifying aphid performance during varying plant growth stages.

Phloem sap quality can differ within a plant, affecting aphid performance [12, 39, 49]. Studies have linked the population growth of soybean aphids with increased nitrogen concentration in the phloem [54, 55]. Following this hypothesis, we found that sucrose and free amino acid concentrations were significantly higher in vascular sap-enriched exudates collected from stems than in exudates from leaf petioles. Moreover, certain amino acids, such as asparagine, threonine, leucine, alanine, phenylalanine, and proline, were significantly higher in stem exudates than leaf petiole exudates. Previous research found that asparagine, glutamic acid, glutamine, glycine, and valine were critical for soybean aphid development and fecundity. Diets with lower than normal levels of these amino acids cause longer development times and lower fecundity with significantly fewer soybean aphids maturing to adulthood [33]. Conversely, green peach aphids (*M*. *persicae*) reared on diets supplemented with asparagine display enhanced growth [12]. Alanine, leucine, and glutamic acid accounted for 43% of variations in the intrinsic rate of increase in populations of the green peach aphids and cabbage aphid (*Brevicoryne brassicae*) [56]. In artificial feeding assays, the population of aphids reared on stem exudates was higher than artificial diet alone and on diet supplemented with leaf petiole exudates mirroring whole plant assays’ results.

Our results demonstrate that the aphid performances on different plant parts are linked to the accessibility and quality of phloem sap. Future transcriptomic and metabolomic studies focused on the sieve element and phloem sap may help understand global changes in phloem chemistry to help address mechanisms underlining aphid performance on different plant parts. Overall, our findings provide insights into how plant physiology affects within-plant aphid population growth, and these results may be useful in developing management plans for soybean aphids.

## Supporting information

S1 Fig Aphid feeding behaviors vary in response to host surface. Electrical penetration graph (EPG) was utilized to determine probing behavior on the adaxial, the abaxial, and the stem surfaces in 8 h of recording time. Values are averages of 30, 33, and 25 samples for the adaxial leaf, abaxial leaf and stem surfaces, respectively. Error bars represent standard error of the mean (S.E.M.).
(TIF)

S1 Table Transitional events observed for each waveform in aphids feeding from the three host surfaces. One-way ANOVA was used to analyze the effects of treatments on each parameter. ^a^ *P*-values that are significant are highlighted in bold. ^b^ The degrees of freedom vary for the different transitions since not all aphids displayed the same transitions.
(DOCX)
