# Supplementary figures and images for: Location, location, location: Feeding site affects aphid performance by altering access and quality of nutrients
Source: PLoS One. 2021 Feb 4;16(2):e0245380. doi: 10.1371/journal.pone.0245380 (PMC7861455; doi:10.1371/journal.pone.0245380)

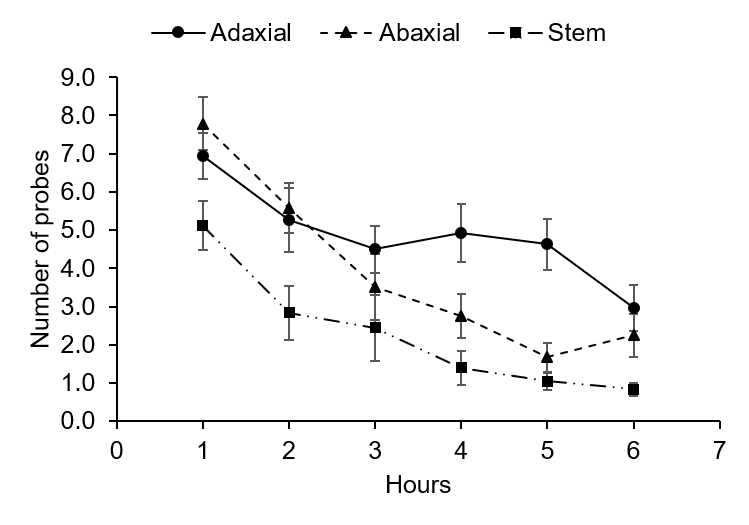

Supplement: S1 Fig — Electrical penetration graph (EPG) was utilized to determine probing behavior on the adaxial, the abaxial, and the stem surfaces in 8 h of recording time. Values are averages of 30, 33, and 25 samples for the adaxial leaf, abaxial leaf and stem surfaces, respectively. Error bars represent standard error of the mean (S.E.M.). (TIF) [file pone.0245380.s001.tif]
